# Genome wide gene expression analysis of nasal mucosa from patients with chronic rhinosinusitis and nasal polyposis stimulated with staphylococcal enterotoxin B

**DOI:** 10.1186/2045-7022-5-S4-O7

**Published:** 2015-06-26

**Authors:** Claudina Perez Novo, Camilla Rydberg Millrud, Natalie De Ruyck, Gabriele Holtappels, Koen Van Crombruggen, Lars Olaf Cardell, Claus Bachert

**Affiliations:** 1 Ghent University, ENT Department, Ghent, Belgium; 2 Karolinska Institute, ENT Department, Stockholm, Sweden

## Introduction

Colonization of the nasal mucosal with *Staphylococcus aureus* and the production of superantigenic enterotoxins by the bacteria are crucial amplifying factors of the pro-inflammatory mechanisms operating in chronic upper airways diseases such as chronic rhinosinusitis and nasal polyposis.

## Methods

Nasal polyp tissue from 10 patients with chronic rhinosinusitis/nasal polyposis and inferior turbinate from 8 healthy subjects were obtained fragmented and cultured in presence and absence of *S. aureus* enterotoxin B (0.5µg/ml) for 24 hours. Then total RNA was isolated, transcribed to cDNA and used for microarray analysis using the Affymetrix Human Gene 2.1 ST Array. Data was then corrected and differentially expressed genes were selected according to the corrected P value < 0.05 and a change fold higher than 2. Pathway overrepresentation analysis was then performed in each group of genes.

## Results

The number of genes showing differential expression between nasal polyp and control tissues as well as the genes and pathways differentially regulated after stimulation with S. aureus enterotoxin B in each of the groups is described in Table 1.

## Conclusions

We demonstrated that *S. aureus* enterotoxin B may influence important pathways linked to T-cell receptor, interferon signalling and adaptative immune response.

